# Leveraging 3D chemical similarity, target and phenotypic data in the identification of drug-protein and drug-adverse effect associations

**DOI:** 10.1186/s13321-016-0147-1

**Published:** 2016-07-01

**Authors:** Santiago Vilar, George Hripcsak

**Affiliations:** Department of Biomedical Informatics, Columbia University Medical Center, New York, NY USA

**Keywords:** 3D molecular structure, Pharmacophoric, Target, Adverse effect

## Abstract

**Background:**

Drug-target identification is crucial to discover novel applications for existing drugs and provide more insights about mechanisms of biological actions, such as adverse drug effects (ADEs). Computational methods along with the integration of current big data sources provide a useful framework for drug-target and drug-adverse effect discovery.

**Results:**

In this article, we propose a method based on the integration of 3D chemical similarity, target and adverse effect data to generate a drug-target-adverse effect predictor along with a simple leveraging system to improve identification of drug-targets and drug-adverse effects. In the first step, we generated a system for multiple drug-target identification based on the application of 3D drug similarity into a large target dataset extracted from the ChEMBL. Next, we developed a target-adverse effect predictor combining targets from ChEMBL with phenotypic information provided by SIDER data source. Both modules were linked to generate a final predictor that establishes hypothesis about new drug-target-adverse effect candidates. Additionally, we showed that leveraging drug-target candidates with phenotypic data is very useful to improve the identification of drug-targets. The integration of phenotypic data into drug-target candidates yielded up to twofold precision improvement. In the opposite direction, leveraging drug-phenotype candidates with target data also yielded a significant enhancement in the performance.

**Conclusions:**

The modeling described in the current study is simple and efficient and has applications at large scale in drug repurposing and drug safety through the identification of mechanism of action of biological effects.

**Electronic supplementary material:**

The online version of this article (doi:10.1186/s13321-016-0147-1) contains supplementary material, which is available to authorized users.

## Background

Drugs can bind different protein targets in the human organism. This action in multiple targets is responsible for therapeutic effects along with clinical adverse effects. For this reason, improvement in the identification of drug-target interactions is of great importance in the discovery of additional applications for drugs already in the market, also called drug repurposing, and in drug safety through the explanation of undesirable adverse effects caused by drugs administration. From the initial discovery stages to the final approval in the pharmaceutical market, molecules have to pass through many evaluation steps with the consequent high associated costs and failure risks [1]. The estimated cost to develop a new drug until commercialization can reach 1 billion [2, 3]. However, drug repurposing strategies can decrease the overall time and cost since existing drugs have been already studied from the point of view of safety and pharmacokinetic profiles [4]. Discovery of new targets for existing drugs is also important in drug safety since supplies valuable information about possible mechanism of action of adverse drug effects (ADEs) [5].

In the last years, different computational methods have been developed to discover new drug-protein interactions [6]. Molecular similarity has been widely applied in medicinal chemistry to discover molecules that bind a specific target [7]. However, similarity can be determined using different measurements. Molecules can be compared based on their 2D molecular structure [8, 9]. Keiser et al. [10] showed the usefulness of comparing molecular fingerprints to generate an approach called SEA (Similarity Ensemble Approach) with great potential in the prediction of new targets. The authors showed that targets can be predicted according to the similarity based on their ligands and discovered new potential applications for existing drugs [10, 11]. On the other hand, 3D molecular structure comparison offers also great potential in medicinal chemistry and drug discovery [12, 13]. It has been shown that both 3D and 2D molecular structure analysis provide different abilities to capture diverse structural patterns related with biological activities [14, 15]. Other types of molecular similarities have also provided great insights in drug-target discovery. Campillos et al. [16] used adverse drug reactions profiles to develop a target identification model validated experimentally. Nevertheless, exploiting clinical data of the disease constitutes another example of a system to identify new targets related to drugs [2]. Some bioinformatics methodologies compared drugs based on gene expression profiles in microarrays and yielded associations between drugs, targets, pathways and diseases [17–21]. Integration of heterogeneous chemical and biological data into predictive models was also a successful strategy in the detection of new targets, indications and adverse effects [22–26]. In summary, different similarity measures and methods have been published with important applications in drug-target identification and hence, drug repurposing and drug safety [27].

On the other hand, drug similarity has also been applied to identify directly associations between drugs and adverse effects. As an example, 2D and 3D structure similarity modeling was previously implemented in the detection of drug candidates responsible for adverse effects [28–30]. Other types of studies with great applications in drug safety and pharmacovigilance have shown potential in drug-adverse effect detection through data mining of the scientific literature [31] or pharmacovilance databases [32–34], such as Electronic Health Records or the FDA Adverse Event Reporting System [35]. The availability of big heterogeneous data sources combined with the explosion of computational methods encourages the large-scale study of relationships between drugs, targets and adverse effects.

**Fig. 1:**
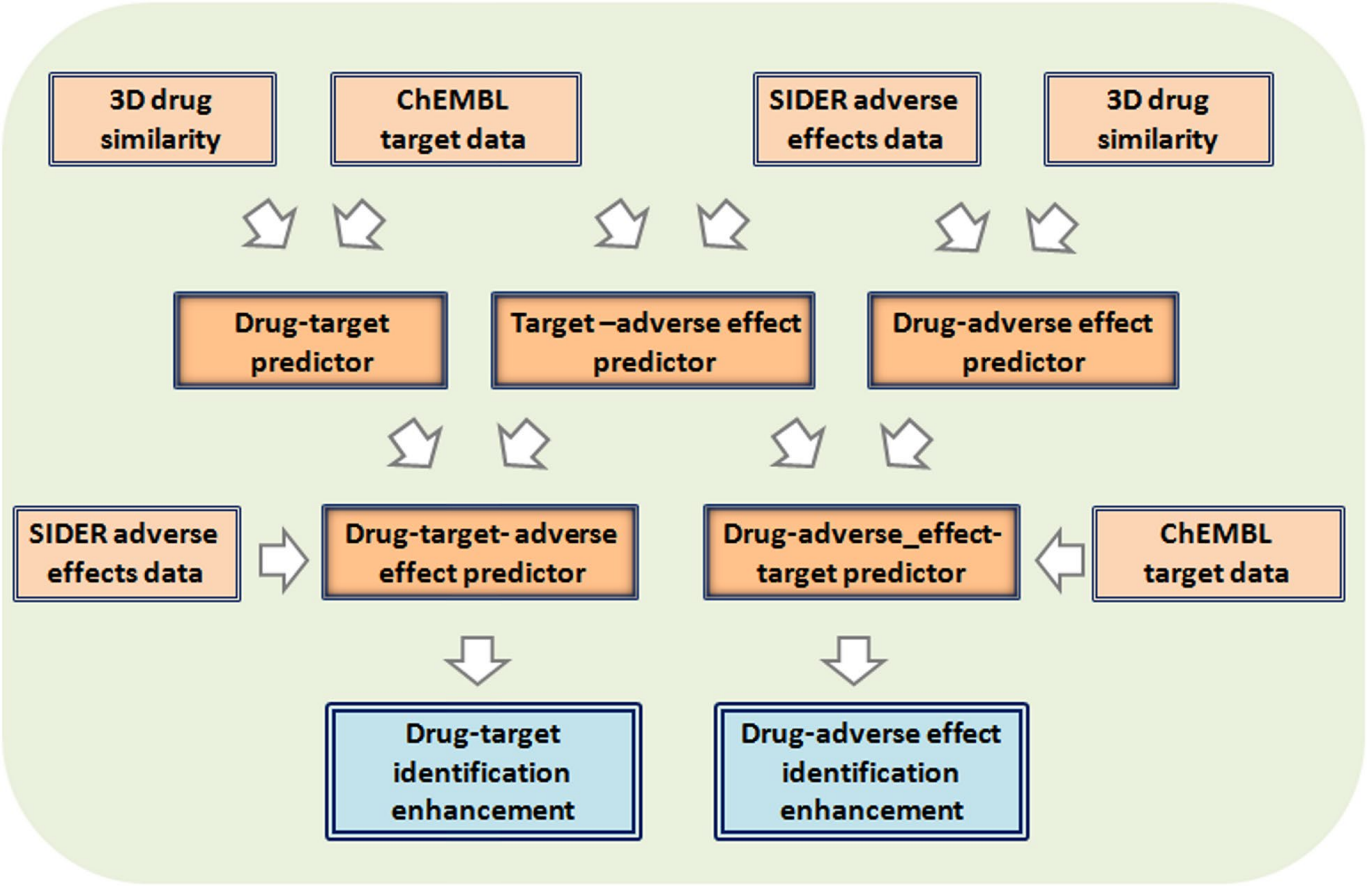
Flowchart of the main steps included in the study

In this article, we integrated and leveraged information from different sources, such as chemical similarity, targets and adverse drug effects (ADEs), to generate a predictor to identify drug-targets, target-adverse effects, and drug-adverse effects associations. We compared drug similarity through a 3D pharmacophoric approach and incorporated similarity data into an extensive source of targets provided by ChEMBL [36] to develop a multi drug-target predictor. Additionally, we developed a target-adverse effect model to be applied to the drug-target predictor with two purposes: to generate hypothesis about drugs, targets and adverse effects, and improve drug-target identification. We hypothesize that a new target predicted for a drug is more likely to be true when: (1) the new target is also associated with adverse effects according to the target-ADE model and (2) the drug is described to be related to those adverse effects in SIDER (reference standard for drug-ADEs) [37]. The same idea but in the opposite direction can be applied in the identification of new drug-ADEs through drug-target leveraging. We also linked the target-adverse effect model to a 3D drug-ADE predictor previously published by our research group [30]. The new predictor was integrated with drug-target data extracted from ChEMBL (reference standard of drug-targets) [36] to improve the recognition of drugs that cause adverse effects. Figure 1 shows the main steps summarizing the study.

## Results

### Drug-target modeling

**Fig. 2:**
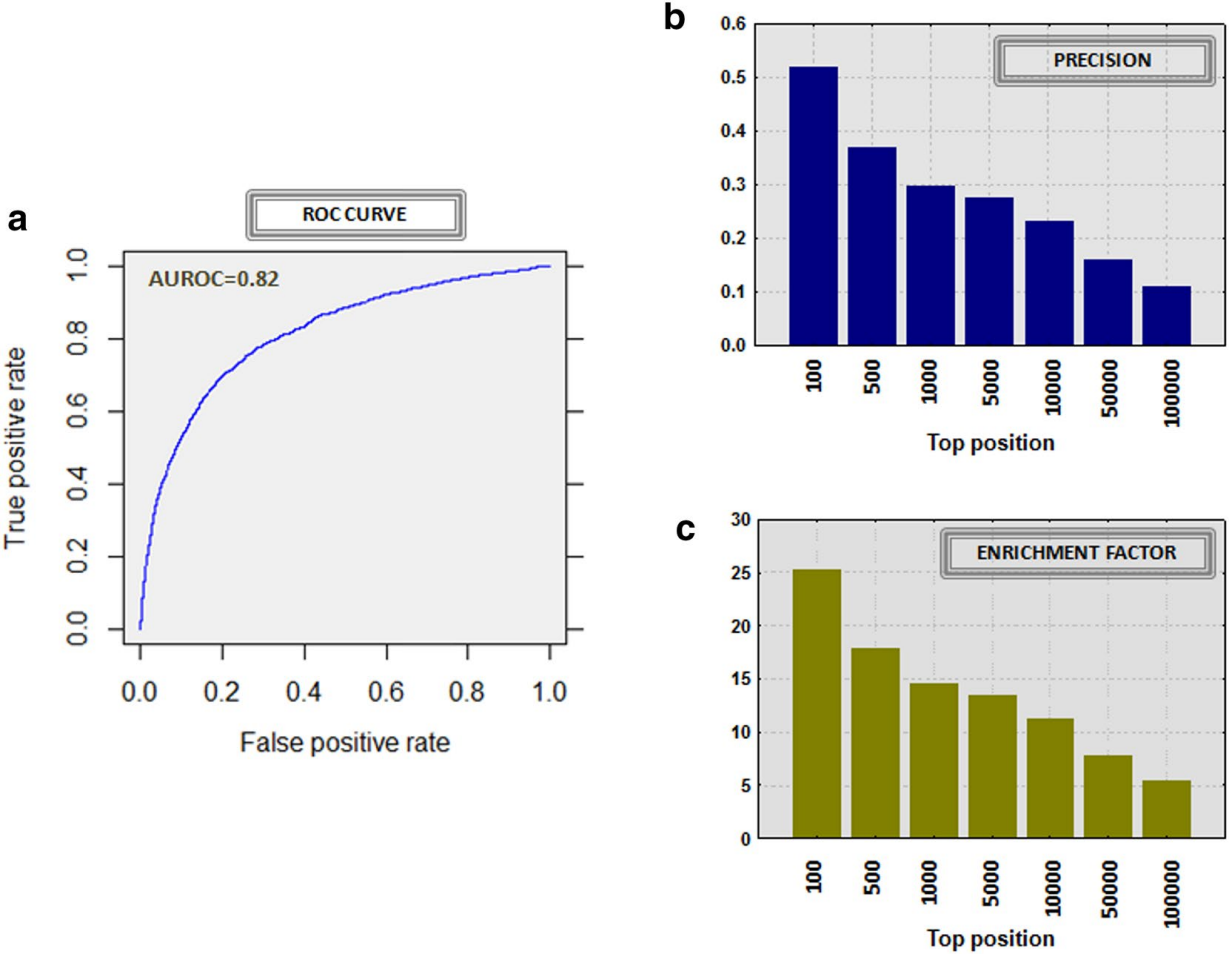
ROC curve (**a**) for the global drug-target predictor along with precision (**b**) and enrichment factors (**c**) in different top positions

We integrated 3D chemical/pharmacophoric similarity into target data from ChEMBL [36] as described in Methods (1526 drugs and 726 targets). Our predictor generated 1,107,876 drug-target combinations with associated leave-one-out scores. Each drug-target candidate is associated with the 3D maximum similarity score against the set of drugs that interact with the same target according to ChEMBL. We labeled as true positives (TP) the drug-target associations already collected in ChEMBL and as false positives (FP) the rest of possible combinations (we defined the FP cases from the unknown cases with no target information collected in the ChEMBL). ROC curve was plotted with an area of 0.82 (see Fig. 2a). We also plotted precision and enrichment factor (EF) in different top positions for the global drug-target predictor (see Fig. 2b, c).

**Fig. 3:**
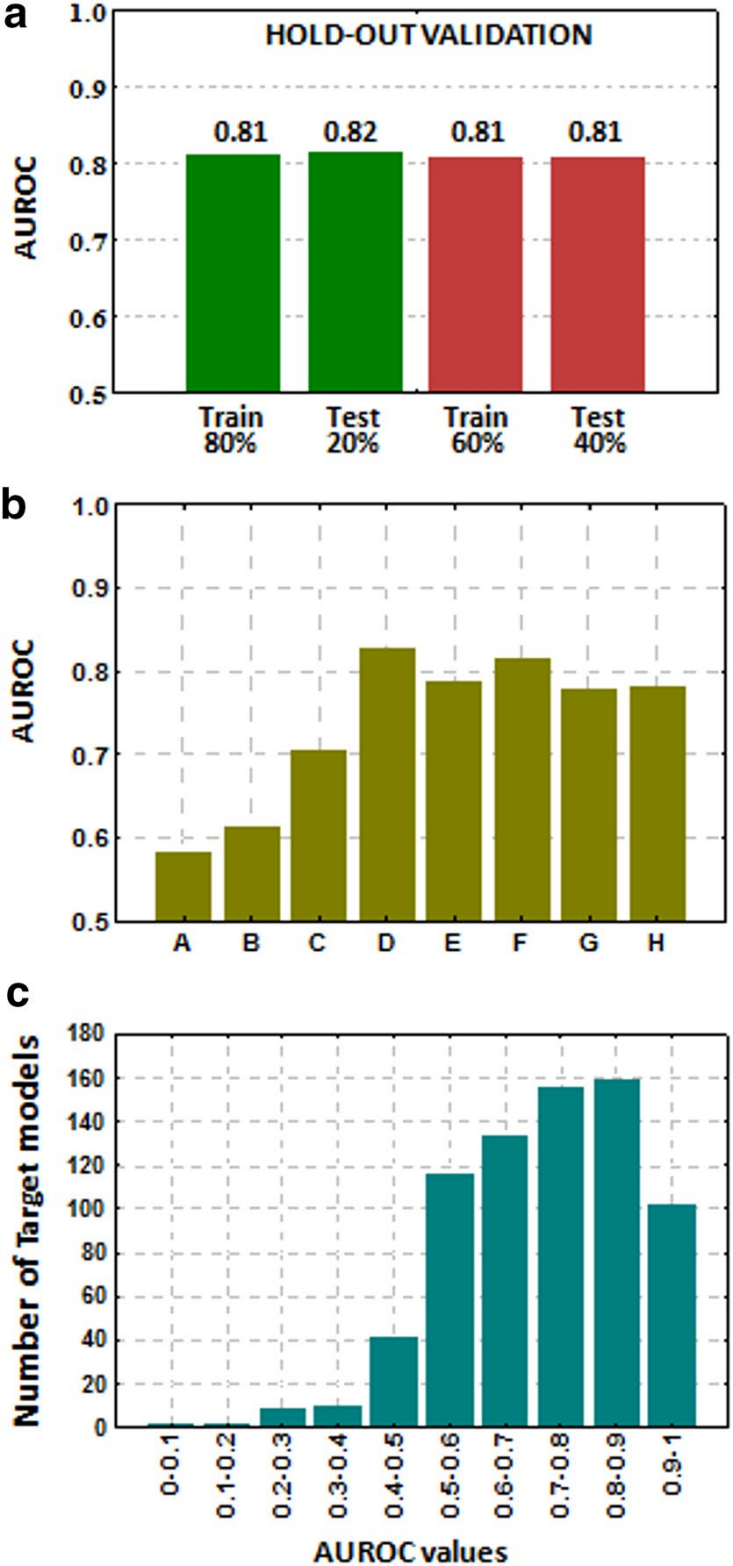
**a** Performance of the 3D drug-target model (AUROCs) in the hold-out validations extracting 20 and 40 % of the initial data into test sets. **b** Performance of the 3D drug-target model (AUROCs) using 8 Anatomical Therapeutic Chemical (ATC) categories as test sets: ACE inhibitors (*A*), Angiotensin II antagonists (*B*), Benzodiazepines (*C*), Beta-blocking agents (*D*), Fluoroquinolones (*E*), Imidazole/triazole derivatives (*F*), Nucleosides/Nucleotides (*G*) and Sulfonamides/urea derivatives (*H*). **c** AUROC values for the individual drug-target models

We carried out a hold-out validation of our predictor. The 20 % of the drug-target combinations found in ChEMBL were included in a test set whereas the model was constructed with the 80 % of the initial drug-target data. This step was repeated but increasing the size of the test to the 40 % and modeling the 60 % of the data. Selection of the sets was made through a random process. Results showed that the predictor barely is affected by the division of the data into training and test sets (see Fig. 3a and Additional file 1: Table S1). Ability of the model to detect novel associations was also assessed and different sets with all the close neighbors were removed. We eliminated from the training all the drugs belonging to 8 Anatomical Therapeutic Chemical (ATC) categories [38], including ACE inhibitors, Angiotensin II antagonists, Benzodiazepines, Beta-blocking agents, Fluoroquinolones, Imidazole/triazole derivatives, Nucleosides/Nucleotides and Sulfonamides. ROC results for each ATC category showed that the model has good ability to predict a class of drugs when no ATC representatives were included to construct the model. The area under the ROC curve (AUROC) for the different groups spans values from 0.58 to 0.83 (see Fig. 3b). Besides performance including all the targets in the ROC (Fig. 2a), we assessed the quality of each individual target model. Figure 3c shows the number of individual target models found for different intervals of AUROCs. Out of 726 individual target models, 427 yielded an AUROC ≥0.70. We did not find correlation between performance in the individual target models and the number of drugs that bind the target in our reference standard (see Additional file 2: Figure S1).

Results for the 3D predictor were compared with a 2D model. Both methods performed similarly and yielded ROC curves greater than 0.80 (see Additional file 3: Figure S2). However, as it was shown previously, 3D structure methods captured a diverse chemical space compared to 2D techniques and can generate different sets of candidates [14, 30, 39]. Previous research showed chemical-biological relationships captured by 3D molecular structure methods and not detected by 2D methods, and vice versa. To prove the potential of detecting a different chemical space, we have plotted in Additional file 4: Figure S3 the 10 % top scored drug–drug similarities in a matrix of drugs using both approaches. Some drug pair examples are detected by 3D methods and not detected according to 2D approaches and vice versa.

### Target-phenotype modeling

**Fig. 4:**
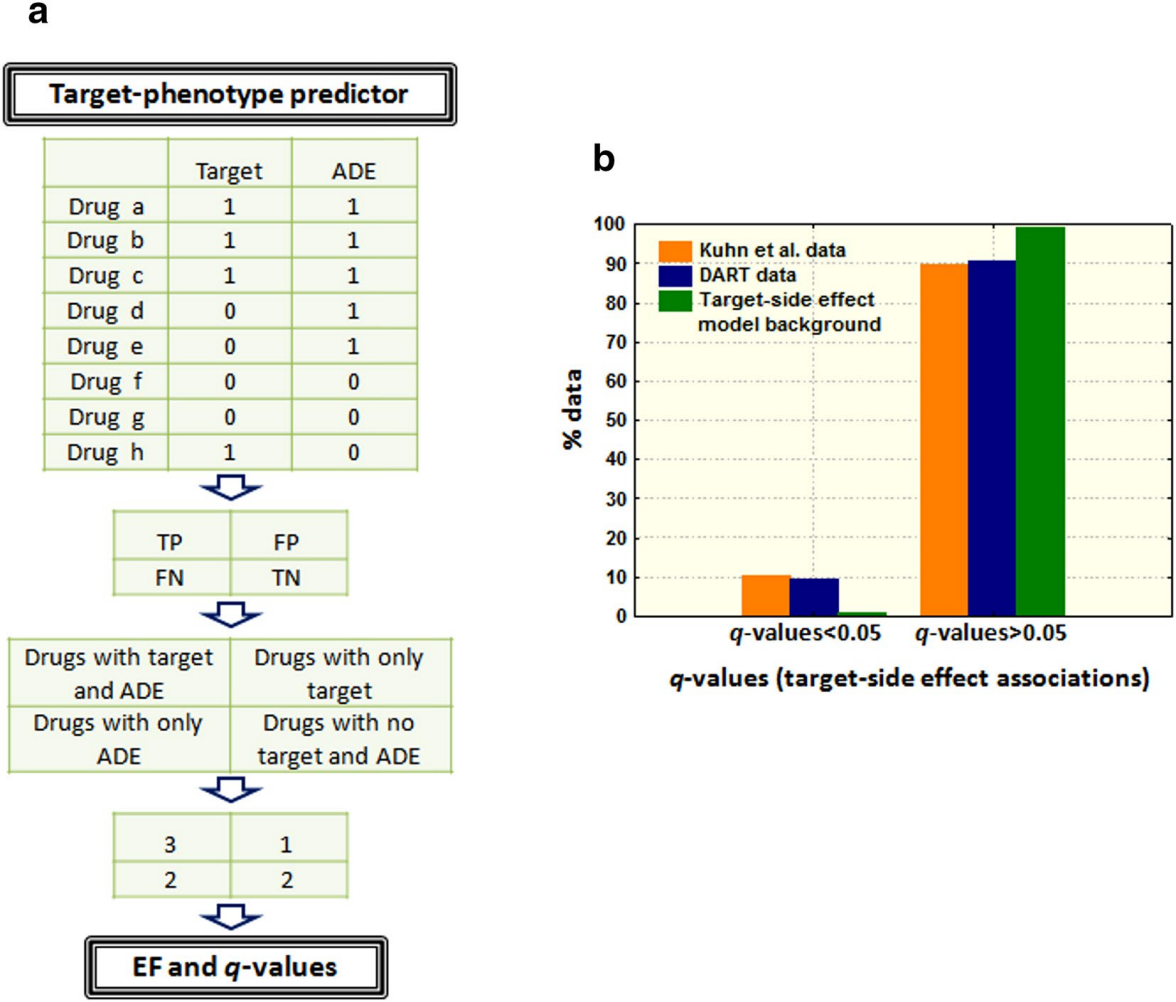
**a** Illustration (no real data) of the target-phenotype predictor. *ADE* Adverse Drug Effect, *EF* Enrichment Factor, *TP* True Positives, *FP* False Positives, *FN* False Negatives, *TN* True Negatives. **b** Validation of the target-adverse effect predictor using two external reference standards of known target-adverse effect associations: a database generated by Kuhn et al. [40] extracted from the literature and manually reviewed, and a set of the associations extracted from DART database. A higher proportion of the target-adverse effect associations in the two reference standards have *q*-values lower than 0.05 compared to the model background

We developed a system to detect targets that have potential to induce adverse reactions. In a similar way as Kuhn et al. [40], we implemented drug-target data extracted from ChEMBL into drug-phenotypic data from SIDER [37] to identify target-adverse effect combinations that are overrepresented. After the removal of targets and adverse effects associated with less than five drugs, we collected a data made out of 347 targets, 1773 adverse effects, 12,341 drug-target cases and 86,397 drug-adverse effect points (Additional file 5: Figure S4 provides number of adverse effects and targets for each drug). We generated causal hypothesis between protein interaction and adverse effects looking for enrichment values of protein associated with adverse effects. Each target-adverse effect combination was associated with an enrichment factor (EF) and a *q*-value (see Fig. 4a and Methods).

The target-phenotype model was validated using two external reference standards of known associations between proteins and adverse reactions. A database generated in a previous study [40] by surveying the scientific literature to find target-adverse effect associations and manually verified was used as a validation set (49 target-adverse effects). A second reference standard of 42 target-adverse effects was taken into account and extracted from the DART database (Drug Adverse Reaction Target Database) [41]. Both test sets are provided in Additional file 6: Table S2. We labeled the known associations as true positives within the whole set generated by our model and calculated the area under the ROC curve for the external tests (AUROCs were 0.70 and 0.71 for the Kuhn and DART tests respectively). More detailed results of our validation process, including sensitivity and specificity at different thresholds, are provided in Additional files 7 and 8: Tables S3 and S4. The *q*-values calculated for the target-adverse effect associations included in the reference standards were lower than the *q*-values in the model background (see Fig. 4b). Our system prioritized the true positive cases over the complete set of target-adverse effect associations. For the next implementation step, a final set of 2426 target-adverse effect candidates was selected with an EF > 5 and *q* < 0.05 and at least 3 drugs in common in both protein and adverse effect (Additional file 9: Table S5 contains the list of 2426 target-adverse effects with EF and *q*-values).

### Linkage of drug-targets and target-adverse effects

**Fig. 5:**
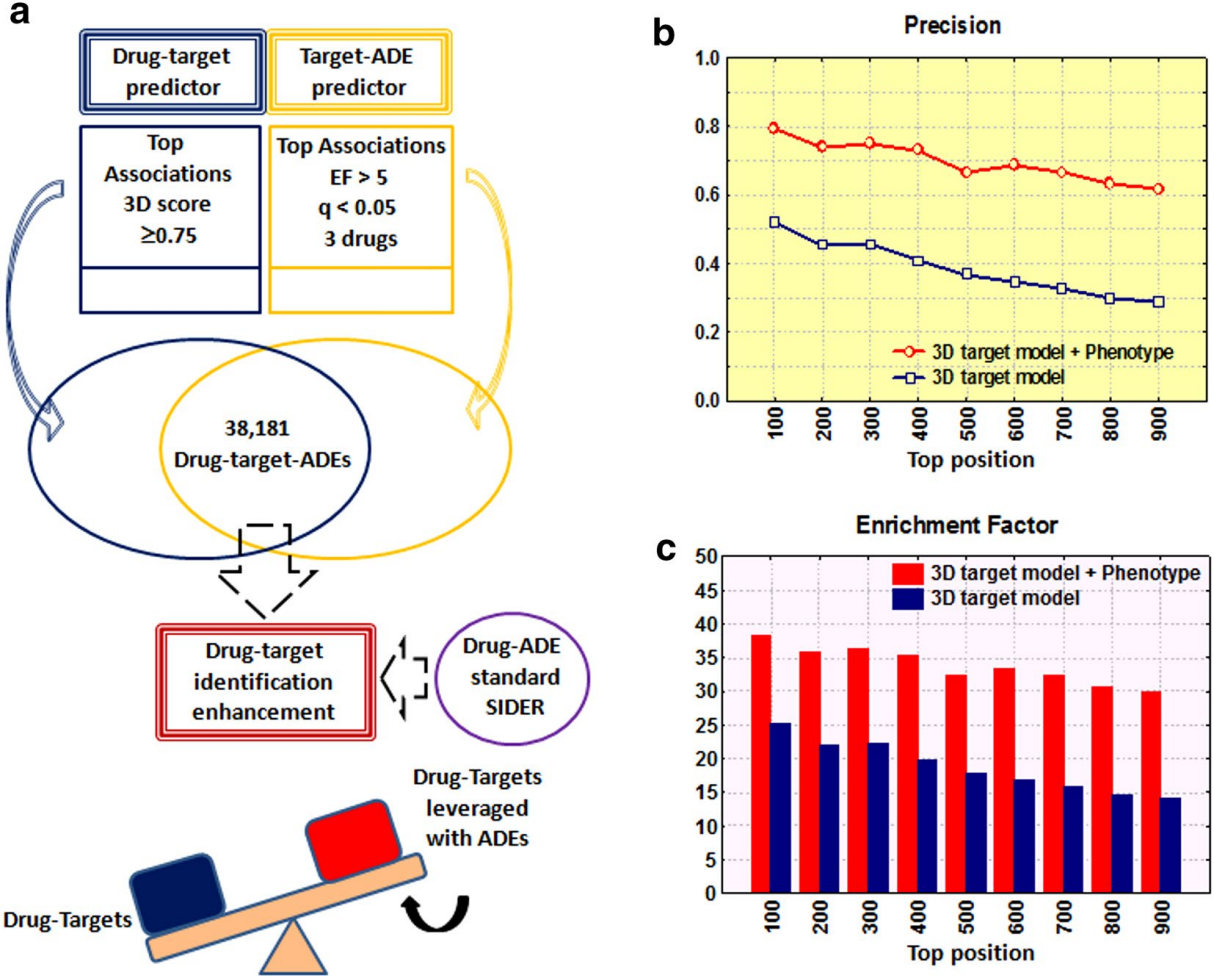
Intersection of drug-target and target-adverse effect data (**a**). Precision (**b**) and Enrichment Factor (**c**) in drug-target identification comparing the 3D drug-target model leveraged with phenotypic data with the 3D drug-target model by itself

The set of target-adverse effects extracted previously, with an EF > 5, *q* < 0.05 and at least 3 drugs representing the case, was linked to each drug-target candidate with a 3D score ≥ 0.75 (see Fig. 5a). It is worth noting that each drug-target candidate can be associated in this way with different adverse effects (ADEs). The predictor generated 38,181 drug-target candidates with multiple associated adverse effect data (338,638 drug-target-adverse effect individual data points are provided in Additional file 10: Table S6). Our database provides drug-target-ADE candidates and further experimental studies would be necessary to confirm or reject the associations.

### Leveraging drug-targets with drug-phenotype

We integrated phenotype data from SIDER into the drug-target associations to improve the performance, what we called leveraging drug-targets with phenotypes. We selected the set of 38,181 drug-target candidates with multiple associated adverse effect data. For each drug-target association, we counted the number of predicted adverse effects corroborated in SIDER for the drug (TP), the number of predicted adverse effects not found in SIDER for the drug (FP), the number of adverse effects described in SIDER not predicted for the drug (FN) and number of adverse effects not described in SIDER nor predicted by the model (TN). Based on these parameters we calculated enrichment factors (EFs) with associated *q*-values for each drug-target association. A set of 921 drug-target associations with an EF > 1 and *q*-value <0.05 was selected for final analysis. When this set of candidates was compared to the initial set of drug-target candidates generated by the 3D model by itself we found an increase in precision and enrichment factor (EF compared against random results). Precision and EF in different top positions comparing both sets are reported in Fig. 5. For instance, the EF reaches values of 32 and 18 at top position 500 calculated with the 3D model with phenotypes and the 3D model by itself respectively. Results showed that integrating drug-phenotype data into the drug-target candidates improved the precision in drug-target identification. Some examples of new drug-target associations yielded by the modeling are shown in Table 1. However, further studies are necessary to confirm the candidates pointed out by the models.

**Table 1:** Examples of some drug-target candidates generated by our predictor

TC^a^	Similar drug in ChEMBL (ATC category)^b^	Drug candidate (ATC category)	3D D-T^c^	Target	EF and *q*-values^d^
0.30	Diclofenac (antiinflammatory agent, non-steroid)	Carbamazepine (carboxamide deriv., antiepileptic)	0.83	Gamma-secretase	EF = 3.17 *q* < .05
0.20	Phenytoin (hydantoin deriv., antiepileptic)	Venlafaxine (antidepressant)	0.82	Aquaporin-4	EF = 2.71 *q* < .05
0.65	Ondansetron (serotonin antagonist, antiemetic-antinauseant)	Molindone (indole deriv., antipsychotic)	0.79	5-HT3 receptor	EF = 17.73 *q* < .05
0.50	Oxymetazoline (descongestant, sympathomimetic)	Molindone (indole deriv., antipsychotic)	0.77	Alpha-2-adrenergic receptor	EF = 22.16 *q* < .05
0.65	Oxybuprocaine (local anesthetic)	Metoclopramide (propulsive)	0.77	DNA repair protein RAD52 homolog	EF = 6.57 *q* < .05
0.39	Niclosamide (salicylic acid deriv., anticestodal)	Thalidomide (immunosuppressant)	0.76	Tyrosine-protein kinase SRC	EF = 2.75 *q* < .05
0.41	Diethyltryptamine (psychedelic drug)	Metoclopramide (propulsive)	0.75	5-HT6 receptor	EF = 8.21 *q* < .05
0.35	Pentamidine (agent against Leishmaniasis/Trypanosomiasis)	Haloperidol (antipsychotic, butyrophenone deriv.)	0.75	Muscarinic acetylcholine M4	EF = 11.22 *q* < .05

Each drug-target association is predicted to cause different adverse effects confirmed in SIDER through the calculation of the EF and *q*-values [predicted adverse effects corroborated in SIDER (TP), predicted adverse effects not found in SIDER (FP), adverse effects described in SIDER and not predicted (FN), adverse effects not described in SIDER and not predicted by the model (TN)]

^a^TC is the Tanimoto coefficient between both drugs using MACCS fingerprint

^b^Similar drug is the most similar drug binding the target in our ChEMBL data calculated with our 3D model

^c^3D D-T is the 3D score that associates the drug candidate with the target according to our 3D model

^d^Enrichment factor (EF) and *q*-values calculated for each drug-target association based on the integration of phenotype data from SIDER

### Leveraging drug-adverse effects with drug-target data

**Fig. 6:**
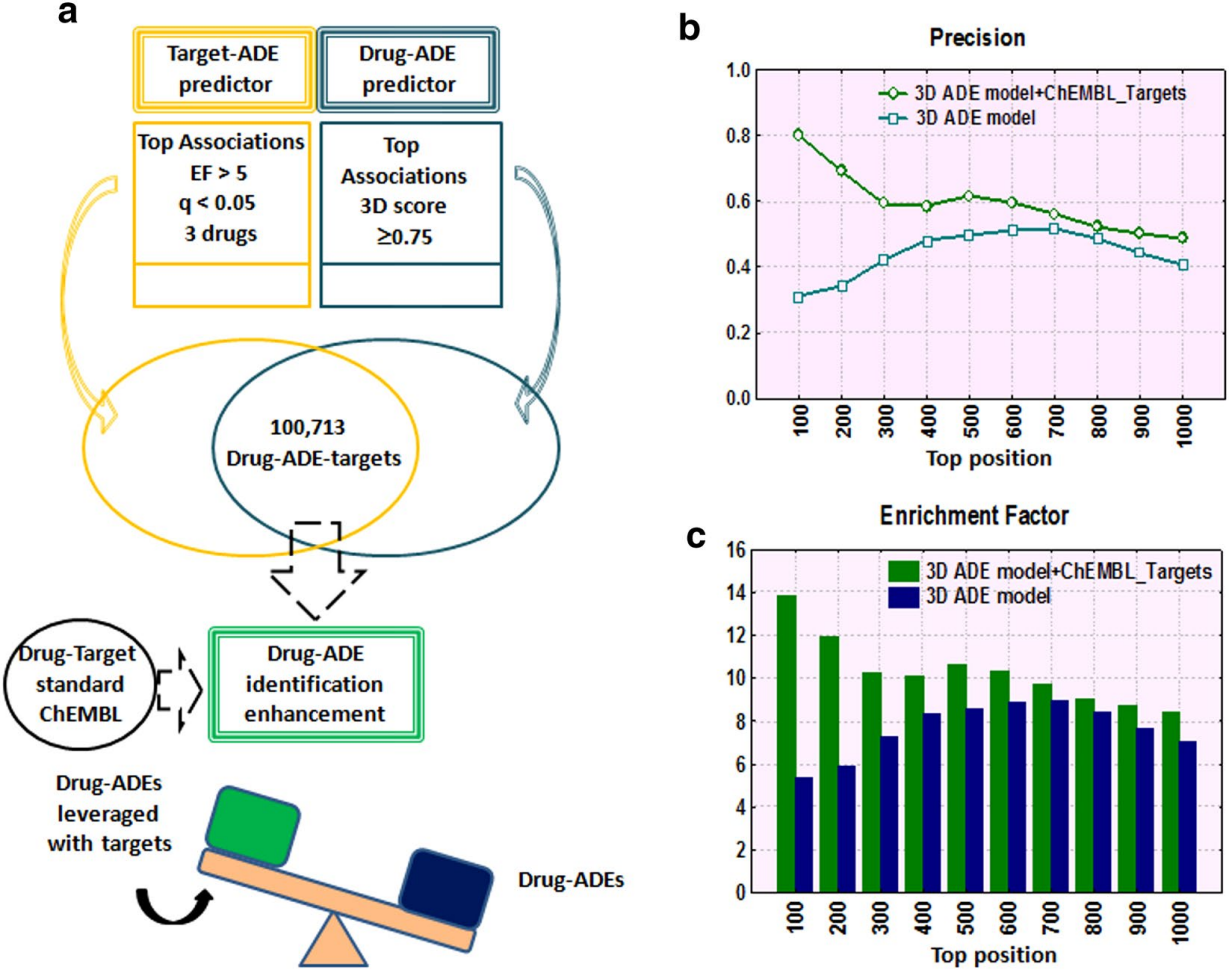
Intersection of drug-adverse effect and target-adverse effect data (**a**). Precision (**b**) and Enrichment Factor (**c**) in drug-adverse effect identification comparing the 3D drug-adverse effect model leveraged with target data with the 3D drug-adverse effect model by itself

In a similar way as described above, we used drug-target data from ChEMBL to improve the identification of drug-adverse effects (see Fig. 6). A previously published 3D drug-adverse effect predictor [30] was used as a source of associations between drugs and adverse effects with a 3D score threshold of 0.75. The drug-adverse effect data was linked to the 2426 target-adverse effect associations and a set of 100,713 drug-adverse effects with associated target data was generated. It is worth noting that for each drug-adverse effect association there can be also different targets associated with the same adverse effect. We calculated EFs and *q*-values for each drug-adverse effect association (see Methods). A set of 1294 drug-adverse effect associations with an EF > 1 and *q*-value <0.05 was extracted as a set of candidates.

We compared the performance of the 3D drug-adverse effect model by itself with the 3D drug-adverse effect model leveraged with target data. Precision and EF in different top positions are shown in Fig. 6b, c. Precision was improved in different top positions when the data is leveraged with target information. However, precision decreases until reach a similar value in the final position 1294 (0.43 for 3D drug-adverse effect model leveraged with phenotypic data and 0.36 for the 3D drug-adverse effect model by itself). Implementing target data into drug-adverse effect candidates enhanced also identification of drug-adverse effect associations.

## Discussion

We have developed a method that integrates 3D structural similarity, protein interactions and adverse effects, in a large scale multi drug-target-adverse effect predictor with novel implications in drug repurposing and patient safety. We also provided a leveraging system to better prioritize the selected drug-target associations through the application of drug-phenotypic data. In the opposite way, improvement in the detection of drug-adverse effects was achieved integrating drug-target data from ChEMBL. We have shown that integrating drug-targets with drug-phenotype data and vice versa is very useful to enhance the performance of the predictors.

Our drug-target predictor scores the candidates based on the maximum 3D similarity against the set of drugs known to bind the protein. This system allows for each drug-target candidate isolating the drug that cause the signaling score and analyze all the information associated, such as type and conditions of the biological assay, protein organism or even different reported activities. The 3D pharmacophoric approach can associate as similar two drugs that belong to the same pharmacological category. However, it also allows the detection of pairs of drugs that are classified in different pharmacological classes. Additional file 11: Figure S5 shows the Anatomical Therapeutic Chemical (ATC) [38] relationship between 1000 random pairs of drugs detected within the threshold of 0.75 for the 3D scoring, along with a histogram of the distribution of the cases. Drugs associated with a high score have the tendency of belonging to the same ATC class. However, as the 3D scores decreases we found more pairs of drugs with different pharmacological profiles.

In the generation of 3D drug similarity data, it is possible to use alternative methodologies, such as different drug conformational analysis, molecular alignments or 3D similarity functions. In our conformational analysis protocol and due to simplicity reasons, only the global minimum energy structure for each drug was retained. However, a more complex approach can be taken into account retaining more conformations for each drug to better represent the bioactive bound conformation. Previous studies by our research group showed that although a set of conformations could describe better the bound form of drugs, the global minimum energy structures yielded also good root mean squared deviations (RMSDs) against crystallized drugs bound to the targets [30]. We collected a set of 158 co-crystallized drug structures in our data from the Protein Data Bank and compared them to: (1) the minimum energy 3D structure generated by our MCMM calculations, (2) the top10 minimum energy conformations extracted from the MCMM (the best RMSD against the crystal is selected). Additional file 12: Figure S6 shows the RMSDs calculated in the comparison. The average RMSD values are 1.66 and 1.05 for both protocols, respectively. Our protocol, taking into account only the minimum energy conformation, is simpler and showed good performance in the recovery of co-crystallized drugs (122 out of 158 presented a RMSD lower than 2.5).

## Methods

### 3D pharmacophoric similarity

#### Drug structures

We downloaded the dataset of drugs available in DrugBank [42]. We did not include proteins, large peptides and drugs with more than 200 atoms due to the complexity to calculate the 3D most stable conformation of molecules with high degree of freedom. DrugBank also provided specified chiral centers information determining bioactive conformation of drugs. Our dataset included 1526 drugs that were pre-processed with LigPrep [43]. This module generated protonation states at neutral pH and a maximum of three enantiomers in the case of lack of chirality information for some centers. Initial molecular geometry was also optimized using OPLS_2005 force field.

#### Monte Carlo Multiple Minimum (MCMM) conformational analysis

We carried out a MCMM conformational analysis for the drugs using Macromodel from Schrödinger [43]. We used water as implicit solvent in the calculation to generate more extended conformations representing with higher fidelity biological active conformations. Non-bonded cut-off distances for H-bond, *van der Waals* and electrostatic forces were set to 4.0, 8.0 and 20.0 Å respectively. Although different minimum energy structures can be studied, we retained only the OPLS_2005 global minimum energy structure as representative of the calculation to simplify next modeling stages.

#### Shape screening

We performed pharmacophoric calculations using Phase from Schrödinger package and assessed 3D similarity for all pairs of drugs. Each drug 3D most stable structure calculated previously was used as a template. Shape screening generated different conformers for the rest of drugs and aligned them to each template to identify common pharmacophoric features between each pair of drugs. The calculation yielded a 3D similarity score, called Phase Sim property that measured the overlapping volume between the same types of pharmacophoric features present in each pair of superimposed drugs. The 3D score spans values between 0 (means minimum 3D similarity) and 1 (means maximum 3D similarity), and it is defined as:

$$ Sim\left( {A,B} \right) = \frac{{{\text{O}}\left( {{\text{A}},{\text{B}}} \right)}}{{\hbox{max} \left( {{\text{O}}\left( {{\text{A}},{\text{A}}} \right),{\text{O}}\left( {{\text{B}},{\text{B}}} \right)} \right)}} $$

where O(A, B) is the overlap of the pharmacophoric sites between drugs A and B and max(O(A, A),O(B, B)) is the maximum of the self-overlaps.

### Target data

We used ChEMBL database [36] as a source of protein data, including pharmacological targets, off-targets, enzymes and transporters. Drugs from DrugBank [42] were mapped to the ChEMBL data using a combination of drug name, InChI keys, and smiles codes resulting in a set of 1526 drugs by which target data was downloaded. Target information in the database was pre-processed as a previous step before data integration in the predictor. This step included incorporation of repeated drug-target cases into a unique case (different bioassays referring to the same target were clustered); elimination of biological data not well specified, such as cases labeled as “not determined”, “not active”, “not tested”, “no inhibition”, “potential missing data”, etc., or drug-target cases with low affinity or potency, i.e. cases where IC_50_, EC_50_ or *K*_i_ was greater than 50 µM. Unspecified cases where the potency was only determined with a threshold,such as “EC_50_ greater than” were also eliminated from the initial data. Additional information, such as assay details was also retained and included in each drug-target case. To increase data robustness, only targets with at least 5 associated drugs were considered in the modeling. Final drug-target data comprised 22,838 drug-target associations (positive controls) with 1526 drugs and 726 targets (1,107,876 possible combinations).

### Phenotypic data

We used SIDER [37] as a resource of 99,423 drug-adverse effect associations (4192 adverse effects related to 996 drugs) extracted from package inserts and public documents. SIDER database is an important source of adverse effect information, although some adverse reactions would need additional confirmation through more studies.

### Drug-target predictor: 3D drug similarity and target integration

Drug similarity based on 3D structure was integrated into the target ChEMBL data through a model that generates all possible drug-target combinations with an associated scoring (3D score). The model compares for each drug the similarity against the set of drugs known to bind each target. If the same drug-target combination is generated in repeated occasions with different scores, i.e., from the comparison of different drug pairs, only the maximum score is retained and the “origin” (drug known to interact with the target and data about potency and assay type) is associated as additional information to the drug-target candidate. In this way each drug-target candidate has associated the maximum similarity score against drugs interacting with the same target in ChEMBL. Out of all the possible drug-target combinations that the predictor generates, some combinations are already found in ChEMBL (positive cases) whereas the other combinations are new associations. ROC curves, precision and enrichment factor (EF) against random results were provided to assess the quality of the predictor:

$$Precision = TP/TP\left( {TP + FP} \right)$$

$$EF = {\frac{{TP}}{{(TP + FP)}}/{\frac{{TPr}}{{(TPr + FPr)}}}}$$

where TP is the number of true positives, FP is the number of false positives and TPr is the number of true positives in a random sample.

### Target-phenotype predictor: target and adverse effect data integration

In a similar way described by Kuhn et al. [40], we integrated drug-phenotypic data from SIDER with drug-target data extracted from ChEMBL to detect overrepresentations of protein-adverse effects (see Fig. 4a). Since the aim is the detection of targets that cause clinical adverse effects, only human proteins in ChEMBL were integrated in SIDER adverse effect data. After mapping our initial 1526 drugs with drugs in SIDER and with drugs with human targets in ChEMBL data, we found 842 drugs by which phenotypic and target data was combined. Targets and adverse effects associated with less than five drugs were not considered in the analysis. Our final data included 347 targets and 1773 adverse effects (615,231 possible target-ADE associations). Enrichment factor (EF) and *p* values (Fisher’s exact test) were calculated for each target-adverse effect combination taking into account number of drugs associated with both target and adverse effect (TP), number of drugs that only bind the target (FP), drugs only associated to the adverse effect (FN), and number of drugs not associated with neither of them (TN). Since multiple associations are taken into account and following the protocol described by Kuhn et al. [40], we addressed multiple hypotheses by using *q*-values calculated with the “qvalue” package in R [44] instead of raw *p*-values. Modeling was validated through the evaluation of two independent test sets of target-adverse effects associations: (1) the Kuhn database, extracted in a previous study [40] from the scientific literature and manually verified and (2) the DART database (Drug Adverse Reaction Target Database) [41]. AUROCs, sensitivity, specificity, precision and enrichment factor at different top thresholds were provided as a comparative measurement.

### Integration of drug-target and target-adverse effect predictors

Final modeling was performed through the integration of previous models, the drug-target and the target-adverse effect predictors. A set of 178,385 drug-target associations with a 3D score threshold of 0.75 was selected as candidates. Regarding the target-adverse effect predictor, we selected 2426 target-adverse effects with EF > 5, *q*-value <0.05 and at least 3 drugs in common in both target and adverse effect. Both sets of signals were intersected to extract a final set of 38,181 drug-targets associated with multiple adverse effects (drug-target-multiADEs). Considering drug-target-adverse effects as unique cases the number of data points is 338,638.

### Leveraging drug-protein interactions with phenotype data

In the set of 38,181 drug-target associations (3D score ≥0.75 and with multiple associated adverse effects), we calculated enrichment factors (EFs) and *q*-values (multiple testing using the “q value” package in R) based on TP (adverse effects corroborated in SIDER for the drug), FP (adverse effects not found in SIDER), FN (adverse effects found in SIDER but not predicted in the modeling), and TN (adverse effects that are not predicted by our model and they are not found in SIDER either). Performance in a set of 921 drug-target associations with an EF > 1 and *q*-value <0.05 was compared to sets extracted from the drug-target model by itself.

### Leveraging drug-adverse effect associations with target data

Associations with a 3D score ≥0.75 between our drugs and adverse effects were extracted from a previous model reported by our research group [30]. In a similar way as described previously, drug-adverse effects were linked to the 2426 target-adverse effect associations to generate a set of 100,713 drug-adverse effects associated to different targets. Enrichment factors (EFs) and *q*-values were calculated for each drug-adverse effect association using target information: TP (predicted targets validated in ChEMBL), FP (predicted targets not validated in ChEMBL), FN (targets present in ChEMBL for the drug that are not predicted by our modeling) and TN (targets not predicted and not described in ChEMBL). A set of 1294 drug-adverse effects with an EF > 1 and *q*-value <0.05 were selected.

## Additional files

10.1186/s13321-016-0147-1 Hold-out validations for the drug-target predictor extracting 20% and 40% of the initial data into test sets.
10.1186/s13321-016-0147-1 AUROC results for each individual target model against the number of drugs that bind the target in our reference standard.
10.1186/s13321-016-0147-1 ROC curves for the drug-target predictors developed with 3D molecular similarity and 2D molecular similarity.
10.1186/s13321-016-0147-1 Overlap in the top 10% scored similarities extracted by the 3D pharmacophoric approach and 2D approach (MACCS).
10.1186/s13321-016-0147-1 Number of side effects and targets for each drug in the target-phenotype model.
10.1186/s13321-016-0147-1 External test sets of target-adverse effect associations.
10.1186/s13321-016-0147-1 AUROC results for the external test sets evaluated with the target-adverse effect model.
10.1186/s13321-016-0147-1 Evaluation of the external sets in the target-phenotype model at different top ranking positions.
10.1186/s13321-016-0147-1 Set of 2,426 target-adverse effects associations extracted from the target-ADE model.
10.1186/s13321-016-0147-1 Dataset with 338,638 drug-target-adverse effect candidates extracted from the modeling.
10.1186/s13321-016-0147-1 Distribution of ATC pairs of drugs through 3D score values and distribution of the number of cases versus 3D score values.
10.1186/s13321-016-0147-1 RMSDs between co-crystallized drugs and theoretical conformations determined through MCMM.

## References

[1] Kola I, Landis J (2004). Can the pharmaceutical industry reduce attrition rates?. Nat Rev Drug Discov.

[2] Dudley JT, Deshpande T, Butte AJ (2011). Exploiting drug-disease relationships for computational drug repositioning. Brief Bioinform.

[3] Adams CP, Brantner VV (2006). Estimating the cost of new drug development: Is it really $802 million?. Health Aff.

[4] Chong CR, Sullivan DJ (2007). New uses for old drugs. Nature.

[5] Lounkine E, Keiser MJ, Whitebread S, Mikhailov D, Hamon J, Jenkins JL, Lavan P, Weber E, Doak AK, Cote S (2012). Large-scale prediction and testing of drug activity on side-effect targets. Nature.

[6] Li J, Zheng S, Chen B, Butte A, Swamidass S, Lu Z (2016). A survey of current trends in computational drug repositioning. Brief Bioinform.

[7] Maggiora G, Vogt M, Stumpfe D, Bajorath J (2014). Molecular similarity in medicinal chemistry. J Med Chem.

[8] Willett P (2011). Similarity searching using 2D structural fingerprints. Methods Mol Biol.

[9] Dimova D, Stumpfe D, Bajorath J (2013). Quantifying the fingerprint descriptor dependence of structure-activity relationship information on a large scale. J Chem Inf Model.

[10] Keiser MJ, Roth BL, Armbruster BN, Ernsberger P, Irwin JJ, Shoichet BK (2007). Relating protein pharmacology by ligand chemistry. Nat Biotechnol.

[11] Keiser MJ, Setola V, Irwin JJ, Laggner C, Abbas AI, Hufeisen SJ, Jensen NH, Kuijer MB, Matos RC, Tran TB (2009). Predicting new molecular targets for known drugs. Nature.

[12] Macchiarulo A, Gioiello A, Thomas C, Massarotti A, Nuti R, Rosatelli E, Sabbatini P, Schoonjans K, Auwerx J, Pellicciari R (2008). Molecular field analysis and 3D-quantitative structure-activity relationship study (MFA 3D-QSAR) unveil novel features of bile acid recognition at TGR5. J Chem Inf Model.

[13] Soderholm AA, Lehtovuori PT, Nyronen TH (2006). Docking and three-dimensional quantitative structure-activity relationship (3D QSAR) analyses of nonsteroidal progesterone receptor ligands. J Med Chem.

[14] Bolton EE, Chen J, Kim S, Han L, He S, Shi W, Simonyan V, Sun Y, Thiessen PA, Wang J (2011). PubChem3D: a new resource for scientists. J Cheminform.

[15] Vilar S, Uriarte E, Santana L, Friedman C, Tatonetti NP (2014). State of the art and development of a drug–drug interaction large scale predictor based on 3D pharmacophoric similarity. Curr Drug Metab.

[16] Campillos M, Kuhn M, Gavin A-C, Jensen LJ, Bork P (2008). Drug target identification using side-effect similarity. Science.

[17] Engreitz JM, Morgan AA, Dudley JT, Chen R, Thathoo R, Altman RB, Butte AJ (2010). Content-based microarray search using differential expression profiles. BMC Bioinform.

[18] Dudley JT, Sirota M, Shenoy M, Pai RK, Roedder S, Chiang AP, Morgan AA, Sarwal MM, Pasricha PJ, Butte AJ (2011). Computational repositioning of the anticonvulsant topiramate for inflammatory bowel disease. Sci Transl Med.

[19] Sirota M, Dudley JT, Kim J, Chiang AP, Morgan AA, Sweet-Cordero A, Sage J, Butte AJ (2011). Discovery and preclinical validation of drug indications using compendia of public gene expression data. Sci Trans Med.

[20] Kunkel SD, Suneja M, Ebert SM, Bongers KS, Fox DK, Malmberg SE, Alipour F, Shields RK, Adams CM (2011). mRNA expression signatures of human skeletal muscle atrophy identify a natural compound that increases muscle mass. Cell Metab.

[21] Iorio F, Bosotti R, Scacheri E, Belcastro V, Mithbaokar P, Ferriero R, Murino L, Tagliaferri R, Brunetti-Pierri N, Isacchi A (2010). Discovery of drug mode of action and drug repositioning from transcriptional responses. Proc Natl Acad Sci USA.

[22] Kuhn M, Campillos M, Gonzalez P, Jensen LJ, Bork P (2008). Large-scale prediction of drug-target relationships. FEBS Lett.

[23] Yamanishi Y, Kotera M, Kanehisa M, Goto S (2010). Drug-target interaction prediction from chemical, genomic and pharmacological data in an integrated framework. Bioinformatics.

[24] Yamanishi Y, Kotera M, Moriya Y, Sawada R, Kanehisa M, Goto S (2014). DINIES: drug-target interaction network inference engine based on supervised analysis. Nucleic Acids Res.

[25] Gottlieb A, Stein GY, Ruppin E, Sharan R (2011). PREDICT: a method for inferring novel drug indications with application to personalized medicine. Mol Syst Biol.

[26] Yamanishi Y, Pauwels E, Kotera M (2012). Drug side-effect prediction based on the integration of chemical and biological spaces. J Chem Inf Model.

[27] Hurle MR, Yang L, Xie Q, Rajpal DK, Sanseau P, Agarwal P (2013). Computational drug repositioning: from data to therapeutics. Clin Pharmacol Ther.

[28] Pauwels E, Stoven V, Yamanishi Y (2011). Predicting drug side-effect profiles: a chemical fragment-based approach. BMC Bioinform.

[29] Vilar S, Ryan PB, Madigan D, Stang PE, Schuemie MJ, Friedman C, Tatonetti NP, Hripcsak G (2014). Similarity-based modeling applied to signal detection in pharmacovigilance. CPT Pharmacomet Syst Pharmacol.

[30] Vilar S, Tatonetti NP, Hripcsak G (2015). 3D pharmacophoric similarity improves multi adverse drug event identification in pharmacovigilance. Sci Rep.

[31] Wang W, Haerian K, Salmasian H, Harpaz R, Chase H, Friedman C (2011). A drug-adverse event extraction algorithm to support pharmacovigilance knowledge mining from PubMed citations. AMIA Annu Symp Proc.

[32] Harpaz R, Perez H, Chase HS, Rabadan R, Hripcsak G, Friedman C (2011). Biclustering of adverse drug events in the FDA’s spontaneous reporting system. Clin Pharmacol Ther.

[33] Harpaz R, Callahan A, Tamang S, Low Y, Odgers D, Finlayson S, Jung K, LePendu P, Shah NH (2014). Text mining for adverse drug events: the promise, challenges, and state of the art. Drug Saf.

[34] Ryan PB, Madigan D, Stang PE, Schuemie MJ, Hripcsak G (2013). Medication-wide association studies. CPT Pharmacomet Syst Pharmacol.

[35] FDA U.S. Food and Drug Administration. FDA Adverse Event Reporting System (FAERS). [http://www.fda.gov/cder/aers/default.htm]

[36] ChEMBL—European Bioinformatics Institute. https://www.ebi.ac.uk/chembl

[37] SIDER Side Effect Resource. http://sideeffects.embl.de

[38] WHO Collaborating Centre for Drug Statistics Methodology. ATC/DDD Index 2015. http://www.whocc.no/atc_ddd_index

[39] Kim S, Bolton EE, Bryant SH (2011). PubChem3D: biologically relevant 3-D similarity. J Cheminform.

[40] Kuhn M, Al Banchaabouchi M, Campillos M, Jensen LJ, Gross C, Gavin A-C, Bork P (2013). Systematic identification of proteins that elicit drug side effects. Mol Syst Biol.

[41] DART: Drug Adverse Reaction Target Database. BIDD Bioinformatics and Drug Design group. http://bidd.nus.edu.sg/group/databases.htm

[42] DrugBank database, version 3.0. http://www.drugbank.ca

[43] Schrödinger package, version 9.2, Schrödinger, LLC, New York, USA, 2011. http://www.schrodinger.com

[44] Storey JD, Tibshirani R (2003). Statistical significance for genome-wide experiments. Proc Natl Acad Sci USA.

