# Supplementary material for: Leveraging 3D chemical similarity, target and phenotypic data in the identification of drug-protein and drug-adverse effect associations
Source: J Cheminform. 2016 Jul 1;8:35. doi: 10.1186/s13321-016-0147-1 (PMC4930585; doi:10.1186/s13321-016-0147-1)
Supplement: Supplementary file 1 — 10.1186/s13321-016-0147-1 Hold-out validations for the drug-target predictor extracting 20% and 40% of the initial data into test sets. [file 13321_2016_147_MOESM1_ESM.docx]

**Table S1.** Hold-out validations for the drug-target predictor extracting 20% and 40% of the initial data into test sets.

| Hold-out validation (train 80% and test 20%) | | | | | | | | |
| --- | --- | --- | --- | --- | --- | --- | --- | --- |
|  |  |  |  |  |  |  |  |  |
| Train 80% | | | | | | | | |
| Position | TP | FP | FN | TN | Sensitivity | Specificity | Precision | EF |
| 100 | 45 | 55 | 18,225 | 1,084,983 | 0.00 | 1.00 | 0.45 | 27.18 |
| 500 | 142 | 358 | 18,128 | 1,084,680 | 0.01 | 1.00 | 0.28 | 17.15 |
| 1,000 | 254 | 746 | 18,016 | 1,084,292 | 0.01 | 1.00 | 0.25 | 15.34 |
| 5,000 | 1,114 | 3,886 | 17,156 | 1,081,152 | 0.06 | 1.00 | 0.22 | 13.45 |
| 10,000 | 1,843 | 8,157 | 16,427 | 1,076,881 | 0.10 | 0.99 | 0.18 | 11.13 |
| 50,000 | 6,294 | 43,706 | 11,976 | 1,041,332 | 0.34 | 0.96 | 0.13 | 7.60 |
| 100,000 | 8,825 | 91,175 | 9,445 | 993,863 | 0.48 | 0.92 | 0.09 | 5.33 |
|  |  |  |  |  |  |  |  |  |
| Test 20% | | | | | | | | |
| Position | TP | FP | FN | TN | Sensitivity | Specificity | Precision | EF |
| 100 | 14 | 86 | 4,554 | 1,084,952 | 0.00 | 1.00 | 0.14 | 33.39 |
| 500 | 36 | 464 | 4,532 | 1,084,574 | 0.01 | 1.00 | 0.07 | 17.17 |
| 1,000 | 65 | 935 | 4,503 | 1,084,103 | 0.01 | 1.00 | 0.07 | 15.50 |
| 5,000 | 302 | 4,698 | 4,266 | 1,080,340 | 0.07 | 1.00 | 0.06 | 14.41 |
| 10,000 | 552 | 9,448 | 4,016 | 1,075,590 | 0.12 | 0.99 | 0.06 | 13.17 |
| 50,000 | 1,620 | 48,380 | 2,948 | 1,036,658 | 0.35 | 0.96 | 0.03 | 7.73 |
| 100,000 | 2,283 | 97,717 | 2,285 | 987,321 | 0.50 | 0.91 | 0.02 | 5.45 |
|  |  |  |  |  |  |  |  |  |
|  |  |  |  |  |  |  |  |  |
| Hold-out validation (train 60% and test 40%) | | | | | | | | |
|  |  |  |  |  |  |  |  |  |
| Train 60% | | | | | | | | |
| Position | TP | FP | FN | TN | Sensitivity | Specificity | Precision | EF |
| 100 | 38 | 62 | 13,665 | 1,084,976 | 0.00 | 1.00 | 0.38 | 30.47 |
| 500 | 97 | 403 | 13,606 | 1,084,635 | 0.01 | 1.00 | 0.19 | 15.56 |
| 1,000 | 182 | 818 | 13,521 | 1,084,220 | 0.01 | 1.00 | 0.18 | 14.59 |
| 5,000 | 880 | 4,120 | 12,823 | 1,080,918 | 0.06 | 1.00 | 0.18 | 14.11 |
| 10,000 | 1,514 | 8,486 | 12,189 | 1,076,552 | 0.11 | 0.99 | 0.15 | 12.14 |
| 50,000 | 4,609 | 45,391 | 9,094 | 1,039,647 | 0.34 | 0.96 | 0.09 | 7.39 |
| 100,000 | 6,519 | 93,481 | 7,184 | 991,557 | 0.48 | 0.91 | 0.07 | 5.23 |
|  |  |  |  |  |  |  |  |  |
| Test 40% | | | | | | | | |
| Position | TP | FP | FN | TN | Sensitivity | Specificity | Precision | EF |
| 100 | 26 | 74 | 9,109 | 1,084,964 | 0.00 | 1.00 | 0.26 | 31.14 |
| 500 | 75 | 425 | 9,060 | 1,084,613 | 0.01 | 1.00 | 0.15 | 17.97 |
| 1,000 | 146 | 854 | 8,989 | 1,084,184 | 0.02 | 1.00 | 0.15 | 17.49 |
| 5,000 | 573 | 4,427 | 8,562 | 1,080,611 | 0.06 | 1.00 | 0.11 | 13.73 |
| 10,000 | 1,019 | 8,981 | 8,116 | 1,076,057 | 0.11 | 0.99 | 0.10 | 12.21 |
| 50,000 | 3,125 | 46,875 | 6,010 | 1,038,163 | 0.34 | 0.96 | 0.06 | 7.49 |
| 100,000 | 4,418 | 95,582 | 4,717 | 989,456 | 0.48 | 0.91 | 0.04 | 5.29 |

TP (true positives), FP (false positives), FN (false negatives), TN (true negatives), EF (enrichment factor).
