# Supplementary material for: Leveraging 3D chemical similarity, target and phenotypic data in the identification of drug-protein and drug-adverse effect associations
Source: J Cheminform. 2016 Jul 1;8:35. doi: 10.1186/s13321-016-0147-1 (PMC4930585; doi:10.1186/s13321-016-0147-1)
Supplement: Supplementary file 2 — 10.1186/s13321-016-0147-1 AUROC results for each individual target model against the number of drugs that bind the target in our reference standard. [file 13321_2016_147_MOESM2_ESM.docx]

**Figure S1.** a) AUROC results for each individual target model (726 target models) against the number of drugs that bind the target in our reference standard (increasing order of number of drugs) b) AUROC results for each individual ADE model (1,780 ADE models) against the number of drugs associated to the ADEs in our reference standard (increasing order of number of drugs) (this model was previously described in *Vilar et al. Sci. Rep. 2015, 5, 8809*).

**
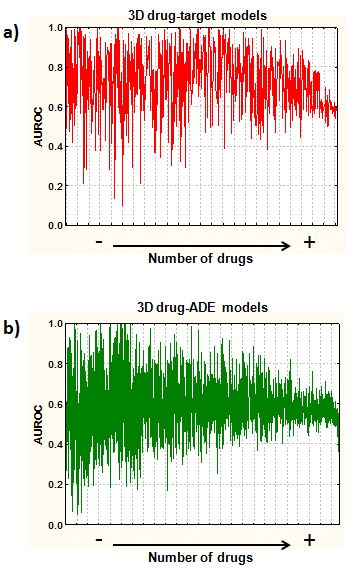
**
