# Supplementary material for: Leveraging 3D chemical similarity, target and phenotypic data in the identification of drug-protein and drug-adverse effect associations
Source: J Cheminform. 2016 Jul 1;8:35. doi: 10.1186/s13321-016-0147-1 (PMC4930585; doi:10.1186/s13321-016-0147-1)

**Figure S2.** ROC curves for the drug-target predictors developed with 3D molecular similarity (red color) and 2D molecular similarity (green color) calculated through MACCS fingerprint. AUROCs are 0.82 and 0.85 respectively.


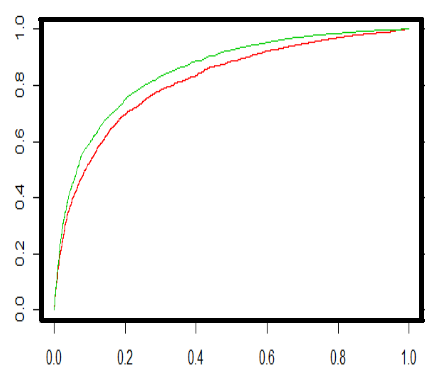

Supplement: Supplementary file 3 — 10.1186/s13321-016-0147-1 ROC curves for the drug-target predictors developed with 3D molecular similarity and 2D molecular similarity. [file 13321_2016_147_MOESM3_ESM.docx]
