# Supplementary material for: Leveraging 3D chemical similarity, target and phenotypic data in the identification of drug-protein and drug-adverse effect associations
Source: J Cheminform. 2016 Jul 1;8:35. doi: 10.1186/s13321-016-0147-1 (PMC4930585; doi:10.1186/s13321-016-0147-1)

**Figure S3.** Overlap in the top 10% scored similarities extracted by the 3D pharmacophoric approach and 2D approach (MACCS). A random set of 25 drugs was selected. Drug pairs retrieved in the top 10% by the 3D approach are represented in red color. Drug pairs detected by the 2D approach are colored in green. Pairs detected by both methods, 2D and 3D, in the top 10% are represented in blue. The matrix is symmetric and the diagonal is colored in black.


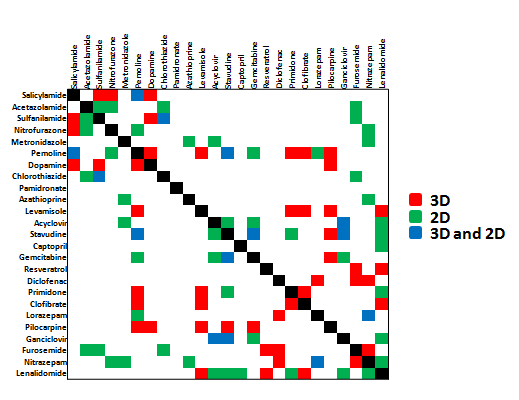

Supplement: Supplementary file 4 — 10.1186/s13321-016-0147-1 Overlap in the top 10% scored similarities extracted by the 3D pharmacophoric approach and 2D approach (MACCS). [file 13321_2016_147_MOESM4_ESM.docx]
