# Supplementary material for: Leveraging 3D chemical similarity, target and phenotypic data in the identification of drug-protein and drug-adverse effect associations
Source: J Cheminform. 2016 Jul 1;8:35. doi: 10.1186/s13321-016-0147-1 (PMC4930585; doi:10.1186/s13321-016-0147-1)

**Figure S4.** Number of side effects and targets for each drug in the target-phenotype model (model with 1,773 side effects and 347 targets). In our data there is no correlation between number of side effects and number of targets.


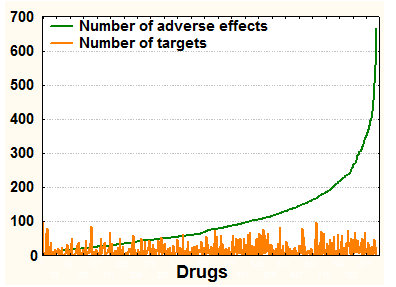

Supplement: Supplementary file 5 — 10.1186/s13321-016-0147-1 Number of side effects and targets for each drug in the target-phenotype model. [file 13321_2016_147_MOESM5_ESM.docx]
