# Supplementary material for: Leveraging 3D chemical similarity, target and phenotypic data in the identification of drug-protein and drug-adverse effect associations
Source: J Cheminform. 2016 Jul 1;8:35. doi: 10.1186/s13321-016-0147-1 (PMC4930585; doi:10.1186/s13321-016-0147-1)
Supplement: Supplementary file 6 — 10.1186/s13321-016-0147-1 External test sets of target-adverse effect associations. [file 13321_2016_147_MOESM6_ESM.docx]

**Table S2.** External test sets of target-adverse effect associations: 49 target-adverse effects from Kuhn data (Mol. Syst. Biol. 9, 663, 2013) and 42 DART target-adverse effects associations. Kuhn data contains 230 target-adverse effects but only 49 were found among the whole set of associations generated by our model and hence, the test set is made out of these 49 target-adverse effects. Out of 253 DART target-adverse effects associations, 42 were found in our data.

| Test set  Kuhn database (protein/adverse effect) | Test set  DART database (protein/adverse effect) |
| --- | --- |
| Serotonin 2a (5-HT2a) receptor/anxiety | Serotonin 2a (5-HT2a) receptor/anxiety |
| Acetylcholinesterase/convulsions | 78 kDa glucose-regulated protein/Gastrointestinal Disorders |
| Androgen Receptor/gynecomastia | Acetylcholinesterase/nausea |
| Androgen Receptor/infertility | Acetylcholinesterase/weakness |
| Androgen Receptor/muscle atrophy | Aldehyde dehydrogenase/renal failure |
| Androgen Receptor/virilization | Alpha-1A adrenergic receptor/hypertension |
| Angiotensin-converting enzyme(ACE)/angioedema | Alpha-2A adrenergic receptor/anxiety |
| Angiotensin-converting enzyme(ACE)/atherosclerotic vascular disease | Alpha-2A adrenergic receptor/edema |
| Angiotensin-converting enzyme(ACE)/cough | Alpha-2A adrenergic receptor/hypertension |
| Angiotensin-converting enzyme(ACE)/hyperkalemia | Androgen Receptor/edema |
| Angiotensin-converting enzyme(ACE)/hypernatremia | Angiotensin-converting enzyme(ACE)/renal failure |
| Angiotensin-converting enzyme(ACE)/hypotension | Beta-1 adrenergic receptor/weakness |
| Canalicular multispecific organic anion transporter 1/bilirubinemia | Cholinesterase/nausea |
| Carbonic anhydrase II/renal tubular acidosis | Cholinesterase/vomiting |
| Cytochrome P450 19A1/flushing | Cyclooxygenase/nausea |
| Cytochrome P450 19A1/osteoporosis | Cyclooxygenase/vomiting |
| Cytochrome P450 19A1/virilization | CYP2C19/sedation |
| Cytochrome P450 2E1/liver disease | CYP2D6/confusion |
| Cytochrome P450 2E1/liver fatty | CYP2D6/nausea |
| D(3) dopamine receptor/tardive dyskinesia | Cytochrome P450 1A2/confusion |
| Epidermal growth factor receptor/diarrhea | Cytochrome P450 1A2/sedation |
| Epidermal growth factor receptor/rash | Cytochrome P450 3A4/confusion |
| Estrogen receptor alpha/osteoporosis | Cytochrome P450 3A4/myopathy |
| Glutamate [NMDA] receptor/seizures | Cytochrome P450 3A4/renal failure |
| Lysosomal alpha-glucosidase/muscle weakness | Cytochrome P450 3A4/sedation |
| Lysosomal alpha-glucosidase/myopathy | D(2) dopamine receptor/sedation |
| Lysosomal alpha-glucosidase/respiratory failure | DNA polymerase beta/nausea |
| Neuronal acetylcholine receptor; alpha4beta2/epilepsy | DNA polymerase beta/peripheral neuropathy |
| Peroxisome proliferator-activated receptor alpha/weight gain | DNA polymerase beta/vomiting |
| Peroxisome proliferator-activated receptor gamma/lipodystrophy | Dopamine D1 receptor/sedation |
| Peroxisome proliferator-activated receptor gamma/obesity | Histamine H1 receptor/sedation |
| Peroxisome proliferator-activated receptor gamma/type 2 diabetes | HMG-CoA reductase/myopathy |
| Serotonin 1a (5-HT1a) receptor/anxiety | Mu opioid receptor/sedation |
| Serotonin 1a (5-HT1a) receptor/hypothermia | Serotonin 1a (5-HT1a) receptor/headache |
| Serotonin 1a (5-HT1a) receptor/mood disorders | Serotonin 1a (5-HT1a) receptor/nausea |
| Serotonin 1a (5-HT1a) receptor/panic disorder | Serotonin 2a (5-HT2a) receptor/hypertension |
| Serotonin 2a (5-HT2a) receptor/bipolar disorder | Serotonin 2a (5-HT2a) receptor/nausea |
| Serotonin 2a (5-HT2a) receptor/tremor | Serotonin 2b (5-HT2b) receptor/anxiety |
| Serotonin 2c (5-HT2c) receptor/anxiety | Serotonin 2b (5-HT2b) receptor/nausea |
| Serotonin 2c (5-HT2c) receptor/weight gain | Serotonin 3 (5-HT3) receptor/anxiety |
| Sodium channel alpha subunits; brain (Types I, II, III)/epilepsy | Serotonin 3 (5-HT3) receptor/nausea |
| Sodium channel alpha subunits; brain (Types I, II, III)/seizures | Serotonin 3 (5-HT3) receptor/vomiting |
| Sodium channel protein type 5 subunit alpha/bundle branch block right |  |
| Sodium channel protein type 5 subunit alpha/heart disease |  |
| Sodium channel protein type 5 subunit alpha/syncope |  |
| Sodium channel protein type 5 subunit alpha/ventricular fibrillation |  |
| Sodium channel protein type 5 subunit alpha/ventricular tachycardia |  |
| Sodium channel protein type II alpha subunit/seizures |  |
| Type-1 angiotensin II receptor/atherosclerotic vascular disease |  |
