# Supplementary material for: Leveraging 3D chemical similarity, target and phenotypic data in the identification of drug-protein and drug-adverse effect associations
Source: J Cheminform. 2016 Jul 1;8:35. doi: 10.1186/s13321-016-0147-1 (PMC4930585; doi:10.1186/s13321-016-0147-1)
Supplement: Supplementary file 7 — 10.1186/s13321-016-0147-1 AUROC results for the external test sets evaluated with the target-adverse effect model. [file 13321_2016_147_MOESM7_ESM.docx]

**Table S3.** AUROC results for the external test sets evaluated with the target-adverse effect model.

|  | Kuhn data | DART data |
| --- | --- | --- |
| Ranking associations using EF | 0.70 | 0.71 |
| Ranking associations using *q*-values | 0.68 | 0.74 |
