# Supplementary material for: Leveraging 3D chemical similarity, target and phenotypic data in the identification of drug-protein and drug-adverse effect associations
Source: J Cheminform. 2016 Jul 1;8:35. doi: 10.1186/s13321-016-0147-1 (PMC4930585; doi:10.1186/s13321-016-0147-1)
Supplement: Supplementary file 8 — 10.1186/s13321-016-0147-1 Evaluation of the external sets in the target-phenotype model at different top ranking positions. [file 13321_2016_147_MOESM8_ESM.docx]

**Table S4.** Evaluation of the external sets (Kuhn et al. and DART data) in the target-phenotype model at different top ranking positions.

| Reference standard: Kuhn et al. database  Ranking associations using enrichment factor | | | | | | | | |
| --- | --- | --- | --- | --- | --- | --- | --- | --- |
| Position | TP | FP | FN | TN | Sensitivity | Specificity | Precision | EF |
| 10,000 | 2 | 9,998 | 47 | 605,184 | 0.04 | 0.98 | 0.0002 | 2.51 |
| 50,000 | 9 | 49,991 | 40 | 565,191 | 0.18 | 0.92 | 0.0002 | 2.26 |
| 100,000 | 14 | 99,986 | 35 | 515,196 | 0.29 | 0.84 | 0.0001 | 1.76 |
| 200,000 | 30 | 199,970 | 19 | 415,212 | 0.61 | 0.67 | 0.0002 | 1.88 |
| 300,000 | 40 | 299,960 | 9 | 315,222 | 0.82 | 0.51 | 0.0001 | 1.67 |
| 400,000 | 45 | 399,955 | 4 | 215,227 | 0.92 | 0.35 | 0.0001 | 1.41 |
| 500,000 | 46 | 499,954 | 3 | 115,228 | 0.94 | 0.19 | 0.0001 | 1.16 |
|  |  |  |  |  |  |  |  |  |
| Reference standard: Kuhn et al. database  Ranking associations using *q*-values | | | | | | | | |
| Position | TP | FP | FN | TN | Sensitivity | Specificity | Precision | EF |
| 10,000 | 9 | 9,991 | 40 | 605,191 | 0.18 | 0.98 | 0.0009 | 11.30 |
| 50,000 | 16 | 49,984 | 33 | 565,198 | 0.33 | 0.92 | 0.0003 | 4.02 |
| 100,000 | 18 | 99,982 | 31 | 515,200 | 0.37 | 0.84 | 0.0002 | 2.26 |
| 200,000 | 29 | 199,971 | 20 | 415,211 | 0.59 | 0.67 | 0.0001 | 1.82 |
| 300,000 | 36 | 299,964 | 13 | 315,218 | 0.73 | 0.51 | 0.0001 | 1.51 |
| 400,000 | 41 | 399,959 | 8 | 215,223 | 0.84 | 0.35 | 0.0001 | 1.29 |
| 500,000 | 44 | 499,956 | 5 | 115,226 | 0.90 | 0.19 | 0.0001 | 1.10 |
|  |  |  |  |  |  |  |  |  |
| Reference standard: DART database  Ranking associations using enrichment factor | | | | | | | | |
| Position | TP | FP | FN | TN | Sensitivity | Specificity | Precision | EF |
| 10,000 | 0 | 10,000 | 42 | 605,189 | 0.00 | 0.98 | 0.0000 | 0.00 |
| 50,000 | 1 | 49,999 | 41 | 565,190 | 0.02 | 0.92 | 0.0000 | 0.25 |
| 100,000 | 6 | 99,994 | 36 | 515,195 | 0.14 | 0.84 | 0.0001 | 0.75 |
| 200,000 | 23 | 199,977 | 19 | 415,212 | 0.55 | 0.67 | 0.0001 | 1.44 |
| 300,000 | 42 | 299,958 | 0 | 315,231 | 1.00 | 0.51 | 0.0001 | 1.76 |
| 400,000 | 42 | 399,958 | 0 | 215,231 | 1.00 | 0.35 | 0.0001 | 1.32 |
| 500,000 | 42 | 499,958 | 0 | 115,231 | 1.00 | 0.19 | 0.0001 | 1.05 |
|  |  |  |  |  |  |  |  |  |
| Reference standard: DART database  Ranking associations using *q*-values | | | | | | | | |
| Position | TP | FP | FN | TN | Sensitivity | Specificity | Precision | EF |
| 10,000 | 8 | 9,992 | 34 | 605,197 | 0.19 | 0.98 | 0.0008 | 10.04 |
| 50,000 | 12 | 49,988 | 30 | 565,201 | 0.29 | 0.92 | 0.0002 | 3.01 |
| 100,000 | 16 | 99,984 | 26 | 515,205 | 0.38 | 0.84 | 0.0002 | 2.01 |
| 200,000 | 26 | 199,974 | 16 | 415,215 | 0.62 | 0.67 | 0.0001 | 1.63 |
| 300,000 | 38 | 299,962 | 4 | 315,227 | 0.90 | 0.51 | 0.0001 | 1.59 |
| 400,000 | 39 | 399,961 | 3 | 215,228 | 0.93 | 0.35 | 0.0001 | 1.22 |
| 500,000 | 39 | 499,961 | 3 | 115,228 | 0.93 | 0.19 | 0.0001 | 0.98 |
