# Supplementary material for: Leveraging 3D chemical similarity, target and phenotypic data in the identification of drug-protein and drug-adverse effect associations
Source: J Cheminform. 2016 Jul 1;8:35. doi: 10.1186/s13321-016-0147-1 (PMC4930585; doi:10.1186/s13321-016-0147-1)

**Figure S5.** a) Distribution of ATC (Anatomical, Therapeutic, Chemical) pairs of drugs through 3D score values. When 3D is high, drugs in the pair belong to the same ATC category (red color - 3 or 4 ATC levels in common). As 3D decreases there are more pairs in different category (blue color - 2 or fewer ATC levels in common). b) Histogram of the distribution of the number of cases versus 3D score values.


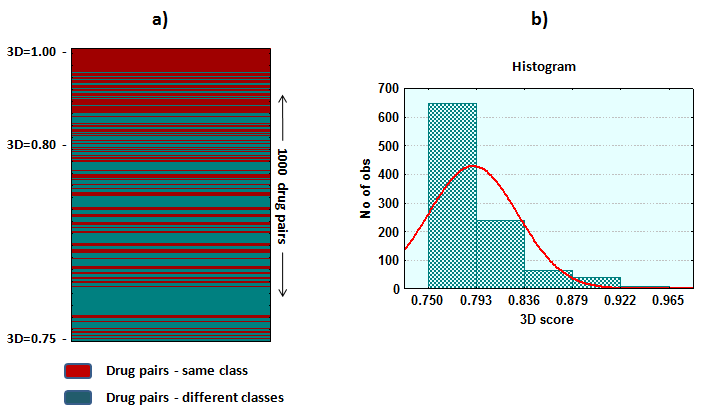

Supplement: Supplementary file 11 — 10.1186/s13321-016-0147-1 Distribution of ATC pairs of drugs through 3D score values and distribution of the number of cases versus 3D score values. [file 13321_2016_147_MOESM11_ESM.docx]
