# Supplementary material for: Leveraging 3D chemical similarity, target and phenotypic data in the identification of drug-protein and drug-adverse effect associations
Source: J Cheminform. 2016 Jul 1;8:35. doi: 10.1186/s13321-016-0147-1 (PMC4930585; doi:10.1186/s13321-016-0147-1)

**Figure S6.** a) RMSD between 158 co-crystallized drugs bound to targets in PDB and theoretical conformations determined through MCMM using two methods: 1) retaining only the minimum energy 3D structure, 2) retaining the top10 minimum energy conformations extracted from the MCMM (the best RMSD against the crystal is selected). The 158 drugs from the PDB are represented in X axis and sorted by molecular complexity (number of atoms).


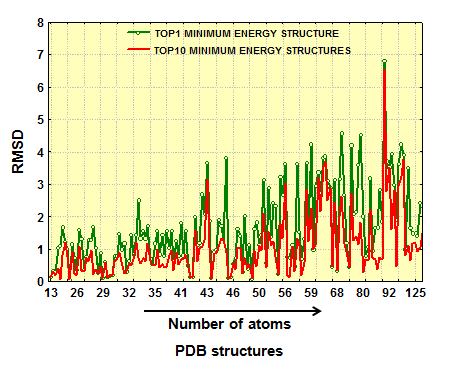

Supplement: Supplementary file 12 — 10.1186/s13321-016-0147-1 RMSDs between co-crystallized drugs and theoretical conformations determined through MCMM. [file 13321_2016_147_MOESM12_ESM.docx]
